# Oncolytic activity of a coxsackievirus B3 strain in human endometrial cancer cell lines

**DOI:** 10.1186/s12985-018-0975-x

**Published:** 2018-04-10

**Authors:** Yanzhen Lin, Wei Wang, Junkai Wan, Ying Yang, Wenkun Fu, Dequan Pan, Linli Cai, Tong Cheng, Xiumin Huang, Yifeng Wang

**Affiliations:** 1 Department of Obstetrics and Gynecology, Zhujiang Hospital, Southern Medical University, Guangzhou, 510282 People’s Republic of China; 2 Department of Obstetrics and Gynecology, Zhongshan Hospital, Xiamen University, Xiamen, 361004 People’s Republic of China; 3 State Key Laboratory of Molecular Vaccinology and Molecular Diagnostics, National Institute of Diagnostics and Vaccine Development in Infectious Diseases, School of Life Science, School of Public Health, Xiamen University, Xiamen, 361102 People’s Republic of China

**Keywords:** Coxsackievirus B3, CV-B3, Oncolytic virus, Endometrial cancer, Virotherapy

## Abstract

**Background:**

Endometrial cancer (EC) is one of the most common gynecological malignancies globally. Although progress has been made in surgical and other adjuvant therapies, there is still a great need to develop new approaches to further reduce the incidence and mortality of EC. Oncolytic virotherapy offers a novel promising option of cancer treatment and has demonstrated good efficacy in preclinical models and clinical trials. However, only few oncolytic viruses have been tested for EC treatment. In this study, the potential of an oncolytic coxsackievirus B3 (CV-B3) strain 2035A (CV-B3/2035A) was investigated as a novel biotherapeutic agent against EC.

**Methods:**

Human EC cell lines (Ishikawa, HEC-1-A and HEC-1-B) were infected with CV-B3/2035A, and viral replication and cytotoxic effects were evaluated in vitro. CV-B3/2035A-induced oncolysis was also investigated in nude mice bearing EC xenografts in vivo and in patient-derived EC samples ex vivo.

**Results:**

Human EC cell lines expressing different levels of CAR and DAF were all susceptible to infection by CV-B3/2035A and supported efficient viral replication in vitro. In the EC xenograft/nude mouse model, both intratumoral and intravenous administrations of CV-B3-2035A exerted significant therapeutic effects against pre-established EC tumors without causing significant treatment-related toxicity and mortality in nude mice. Moreover, CV-B3/2035A treatment resulted in decreased viability of patient-derived EC samples ex vivo.

**Conclusions:**

CV-B3/2035A showed oncolytic activity in human EC cell lines both in vitro and in vivo as well as in patient-derived EC samples ex vivo and thus could be used as an alternative virotherapy agent for the treatment of EC.

**Electronic supplementary material:**

The online version of this article (10.1186/s12985-018-0975-x) contains supplementary material, which is available to authorized users.

## Background

Endometrial cancer (EC) is one of the most common gynaecological cancers in the world [1]. There are two histological types of EC. The most common type is endometrioid adenocarcinoma, or type I EC, and type II ECs are predominantly aggressive carcinomas [2]. Today, the prevalence of EC is increasing due to the increasing number of elderly women and increasing rates of obesity and metabolic syndromes, and thus EC will continue to be a major problem for health-care systems worldwide [1, 3, 4]. Presently for women with early-stage EC, standard management with hysterectomy, bilateral salpingoophorectomy with or without pelvic/para-aortic lymph node dissection confers an excellent 5-year overall survival rate (> 90%) [5–8]. In more advanced cases, surgery can be followed by adjuvant therapies including radiation therapy, chemotherapy or hormone therapy [9–14]. However, current treatments result in low response rates and survival benefit for patients with high-graded, metastatic or recurrent EC [14–16], which calls for the necessity of new innovative therapeutic approaches.

Oncolytic virotherapy has gained increasing interest as a novel immunotherapeutic approach to treat cancers in recent years (reviewed in [17, 18]). Although not fully understood, oncolytic viruses are thought to exert anti-tumor effects by inducing selective tumor cell death, which not only directly eliminates viable tumor cells but also sets the stage for initiation of systemic anti-tumor immune responses. Targeting of these viruses to cancer cells is mainly based on the overexpression of viral entry receptors on tumor cells and aberrant signaling pathways within tumor cells. To date, a number of oncolytic viruses, including adenoviruses, herpesviruses, reoviruses, poxviruses and picornaviruses, have demonstrated appreciable efficacy in preclinical models and clinical trials (reviewed in [17, 18]). A modified herpes simplex virus type 1 (HSV-1), T-VEC from Amgen, is the first and so far only oncolytic virotherapy to be approved for cancer treatment by the US Food and Drug Administration (FDA) [19, 20]. However, only few oncolytic viruses have been tested for EC treatment.

Coxsackievirus B3 (CV-B3) is a small, nonenveloped enterovirus with a single-stranded, positive-stranded RNA genome in the *Picornaviridae* family. Previously, CV-B3 prototype Nancy strain (CV-B3/Nancy) has demonstrated oncolytic activity against various human tumor cell lines including colon carcinoma, pancreatic carcinoma, breast carcinoma, cervical carcinoma, human non–small cell lung cancer (NSCLC), etc. [21] These data indicate that CV-B3 may hold promise as a broad-spectrum oncolytic agent that can infect and kill a wide variety of human tumor cell types, possibly including human EC cells. In the present study, the oncolytic activity of a CV-B3 2035A strain (CV-B3/2035A) was investigated in different human EC cell lines both in vitro and in vivo. Additionally, CV-B3/2035A-induced oncolysis was assessed in patient-derived EC samples ex vivo. The results of this study indicate that CV-B3/2035A could be used as an effective oncolytic and therapeutic agent against human EC.

## Methods

### Cells and virus

Two human EC cell lines (HEC-1-A and Ishikawa) and a human rhabdomyosarcoma cell line (RD) were purchased from the American Type Culture Collection (Manassas, USA). A human EC cell line (HEC-1-B) was purchased from the China Infrastructure of Cell Line Resources (Beijing, China). All cells were maintained in 5% CO_2_ at 37 °C in their respective media as follows: HEC-1-A in McCoy’s 5a medium, HEC-1-B and Ishikawa in Minimum Essential Medium (MEM) and RD in Dulbecco’s modified Eagle’s medium (DMEM) (all from Invitrogen). The culture media used were all supplemented with 10% (*v*/v) fetal bovine serum (FBS), 50 U/ml penicillin, and 50 μg/ml streptomycin (Invitrogen).

CV-B3 2035A strain (GenBank Accession No. KY286529.1) was isolated from a throat swab of a patient with mild hand, foot and mouse disease (HFMD) from the Centers for Disease Control and Prevention (CDC) of Xiamen, China in 2008, and was propagated in RD cells. Virus titers were determined by TCID50 assay on RD cells according to the Reed-Muench method [22], and were used for all multiplicity of infection (MOI) determinations described.

### Flow cytometry analysis

Dispersed EC cells (1 × 10^6^) were incubated with either a mouse monoclonal antibody (mAb) against coxsackievirus and adenovirus receptor (CAR; clone RmCB; Millipore) followed by incubation with fluorescein isothiocyanate (FITC)-conjugated goat anti-mouse secondary antibody (Sigma) or a FITC-conjugated mouse mAb against decay-accelerating factor (DAF; BD Biosciences) for 30 min (or mock treated) at room temperature. Cells were then washed, pelleted, resuspended in phosphate buffered saline (PBS), and analyzed for receptor expression using a flow cytometer (BD FACSAria™ III, BD Bioscience), with data analyzed using FlowJo software (TreeStar, USA).

### In vitro viral infectivity assay

EC cells were seeded in 96-well plates in the appropriate medium without FBS and then infected with ten-fold serial dilutions of CV-B3/2035A (100 μl/well in quadruplicate, from MOI = 10 to MOI = 10^− 3^). After incubation in 5% CO_2_ at 37 °C for 72 h, cytotoxicity was assessed using a Cell Counting Kit-8 (CCK-8) assay according to the manufacture’s instructions (Beyotime Institute of Biotechnology, China).

### In vitro viral growth kinetics in infected tumor cells

EC cells were seeded in 24-well plates and then infected with CV-B3/2035A at an MOI of 0.1 in triplicate and allowed to adsorb for 1 h at 37 °C. Then cells were washed three times with media to remove unbound virus and fresh media were added. Cells were incubated in 5% CO_2_ at 37 °C and harvested at 2, 24 or 48 h post-infection (hpi). The harvested cells and supernatants were freeze-thawed twice and the lysates were centrifuged at 5000 g for 10 min at 4 °C. Virus yield in the obtained supernatants were titrated on RD cells as described above.

### Quantitative RT-PCR (qRT-PCR)

Total RNA was extracted from tissues using a GenMagSpin Viral DNA/RNA Kit (GenMag Bio, China). The qRT-PCR was performed with the One-Step RT-PCR Kit (GenMag Bio, China) following the manufacturer’s instructions in the LightCycler® 96 system (Roche, Switzerland). Primer and probe sequences are shown in Additional file 1: Table S1. The thermal conditions were set to 50 °C for 10 min, 95 °C for 10 min, followed by 45 cycles of 95 °C for 15 s and 55 °C for 50 s. Relative cDNA level was calculated using a standard curve of Ct (cycle threshold) values generated from the dilution series of pMD18-CV-B3/2035A plasmid containing the target gene.

### In vivo anti-tumor studies using subcutaneous xenografts in nude mice

All animal procedures were carried out under specific-pathogen-free (SPF) conditions and in accordance with the approved animal use protocols of Xiamen University Laboratory Animal Center. EC cells (5 × 10^6^ cells) were injected subcutaneously into the right or bilateral flanks of 6–8 week old female nude mice (BALB/c-nu/nu mice; Beijing Vital River Laboratory Animal Technology Co., Ltd., China). Treatment started when tumors reached diameters of ~ 0.5 cm. For evaluating intratumoral use of CV-B3/2035A, the right flank tumors were injected with five consecutive doses (5 × 10^6^ TCID50 per dose) of CV-B3/2035A at 2-day intervals, or with PBS as a negative control. For evaluating intravenous use of CV-B3, nude mice with bilateral EC xenografts were administered with a single dose of 1 × 10^6^ TCID50 of CV-B3/2035A or PBS via lateral tail veins. Tumor growth was monitored and measured with calipers every other day for 20 days. The tumor volume was calculated as length × width × width/2 and expressed as means ± SD. For the biodistribution analysis of CV-B3/2035A, presence of virus in nude mice 2 and 6 days after a single intravenous injection was quantified by qRT-PCR from tissue samples, including tumor, brain, heart, liver, spleen, lung and kidney. For the preliminary toxicity evaluation of CV-B3/2035A, body weight of all groups of nude mice was monitored regularly, and vital tissues of nude mice given a single intravenous injection were histologically analyzed after H&E staining on day 20.

### Histology and immunohistochemistry

Paraffin sections (5 μm thick) of tissues (including tumor, brain, heart, liver, spleen, lung and kidney) of nude mice were deparaffinized in xylene and rehydrated in graded alcohols. For histological analysis, sections of organs of nude mice were stained with hematoxylin and eosin (H&E). For analysis of CV-B3/2035A replication in tumor xenografts, immunohistochemistry (IHC) was performed for viral antigen expression as previously described [23]. Briefly, sections were subjected to heat-induced antigen retrieval using citrate buffer, followed by endogenous peroxidase quenching using hydrogen peroxide. Then, sections were blocked with normal goat serum and incubated with a mouse mAb against CV-B3 (clone L8F12, unpublished). Next, IHC staining was performed using an Ultrasensitive TMS-P kit (Fuzhou Maixin Biotechnology Development Co., Ltd., China) and a DAB detection kit (streptavidin-biotin; Fuzhou Maixin Biotechnology Development Co., Ltd., China) according to the manufacturer’s instructions. Finally, sections were counterstained with hematoxylin, dehydrated and cover-slipped.

### Histoculture drug response assay

Five human EC samples were obtained either during biopsy or surgical excision at hospital between November 2016 and September 2017 (Table 1). The normal endometria were obtained from two patients with benign uterine diseases who underwent surgical hysterectomies. None of the EC patients received any treatment before surgery. Informed consent for this study was obtained from each patient. This study was approved by the Institutional Review Board of the hospital and the Research Ethics Committee of Xiamen University. Experiments were carried out in strict accordance with the guidelines and regulations of Xiamen University.

**Table 1:** Nucleotide and amino acid identities (%) between CV-B3/2035A and CV-B3/Nancy

Region	Position^*^	Versus CV-B3/Nancy
Genome nucleotide	1–7388	77.2
5′ UTR nucleotide	1–742	82.4
P1 nucleotide	743–3304	76.3
P1 amino acid	–	96.9
P2 nucleotide	3305–5029	77.4
P2 amino acid	–	95.7
P3 nucleotide	5030–7297	78.1
P3 amino acid	–	97.7
3′ UTR nucleotide	7298–7388	84.3

*UTR* untranslated region

*Positions are numbered according to CV-B3 strain 2035A

The histoculture drug response assay (HDRA) was used for assessment of the oncolytic ability of CV-B3/2035A in patient-derived tumor samples ex vivo as described elsewhere [24], with slight modifications. Briefly, EC tissues were cut into pieces (~ 1 mm^3^) under sterile condition and placed on 0.5 cm^2^ filter papers, which were not completely submerged in DMEM containing 15% FBS, 100 U/ml penicillin and 100 μg/ml streptomycin, in equal quantities in each well of 24-well plates. After overnight culture at 37 °C, EC tissues were exposed to 10^6^ TCID50 of CV-B3/2035A per well or left untreated. Following incubation for 72 h, 100 μl of 0.06% collagenase (type IV; Sigma) in DMEM was added to each well and the plates were incubated at 37 °C for another 4 h. Next, tissues and supernatants from each well were harvested and centrifuged at 5000 g for 10 min at 4 °C. The resultant pellets from each well were resuspended in 100 μl media and cell viability was assessed by CCK-8 assay in 96-well plates. The inhibition rate was calculated as: Inhibition rate (%) = (1 – A/B) × 100, where A is the mean absorbance of the treated wells per 1 g of tumor and B is the mean absorbance of the control wells per 1 g of tumor.

### Statistical analysis

All statistical analysis were done using GraphPad Prism 7 (GraphPad Software Inc., USA). Comparisons of viral titers were performed using unpaired Student t-test. Comparisons of tumor growth curves and body weight curves between the CV-B3-treatment groups and control groups were performed using two-way repeated measures ANOVA.

## Results

### Oncolytic activity of CV-B3/2035A in human EC cell lines in vitro

**Fig. 1:**
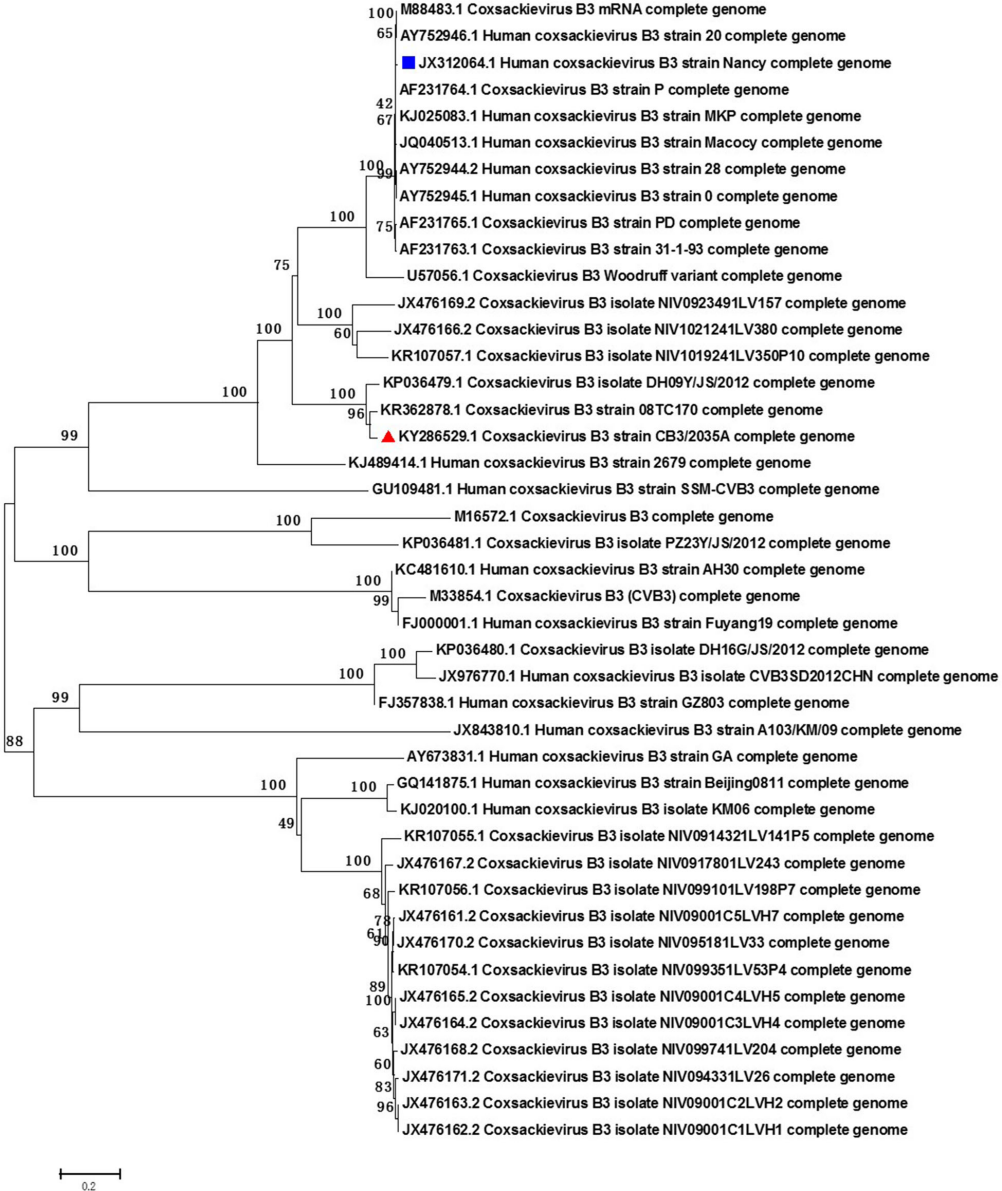
Phylogenetic tree showing relationships between the complete genomes of CV-B3 2035A strain and the other 42 CV-B3 strains. CV-B3/2035A is indicated by a red marker, while a recently reported oncolytic strain, CV-B3/Nancy is indicated by a blue marker

**Fig. 2:**
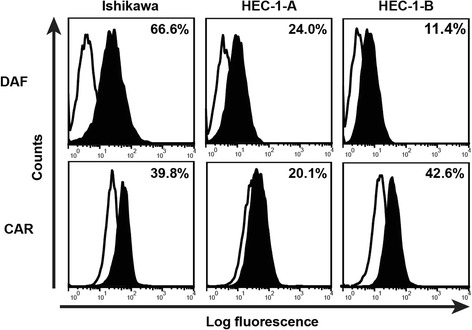
Flow cytometric analysis of surface CAR and DAF expression on EC cells. Open and closed histograms represent the measured fluorescence of cells incubated with an isotype control antibody and anti-DAF or anti-CAR antibody, respectively. The percentage of the DAF- or CAR-positive cells is indicated in each panel

**Fig. 3:**
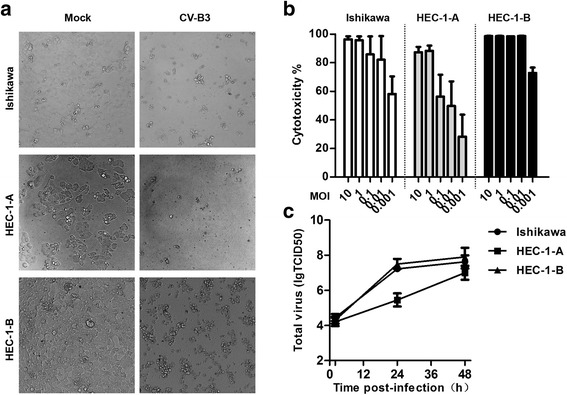
CV-B3/2035A-induced lytic infection of EC cells in vitro. **a** EC cell monolayers were either mock-infected or infected with CV-B3/2035A at an MOI of 10, and photomicrographs were taken at 72 h post-infection (× 20 original magnification), respectively. **b** EC cells in 96-well plates were infected with tenfold serial dilutions of CV-B3/2035A (starting MOI = 10). Following incubation at 37 °C for 72 h, cytotoxicity was measured using CCK8 assay. The mean percentage cytotoxicity (±SD) is shown from quadruplicate wells from one representative experiment. **c** Growth kinetics of CV-B3/2035A in EC cells in vitro. CV-B3/2035A was inoculated in triplicate onto monolayers of EC cells in 24-well plates at an MOI of 0.1. After removal of unbound virus, the cells were incubated at 37 °C and virus yield was determined at selected time points (2, 24 and 48 h) after freezing-thawing by TCID50 assay on RD cells

CV-B3 2035A strain was isolated from a throat swab of an HFMD patient in Xiamen, China in 2008. A phylogenetic tree was constructed for CV-B3/2035A and 42 other CV-B3 strains based on the whole genome sequences (Fig. 1). Sequence comparison and phylogenetic analysis showed that CV-B3/2035A had a low level of nucleotide sequence identity to the prototype oncolytic CV-B3/Nancy (Table 1). On the other hand, similar to CV-B3/Nancy [21], CV-B3/2035A exhibited oncolytic activities against various human tumor cell types (detailed information shown in Additional file 1: Table S2). For assessment of the oncolytic potential of CV-B3/2035A against human EC, Ishikawa cells and HEC-1-A/HEC-1-B cells were used as models of type I and type II EC, respectively, as previously reported [25, 26]. Firstly, the expression levels of the CV-B3 receptors, CAR and DAF, on the EC cell lines were evaluated by flow cytometry. CAR, in particular, is a major entry receptor for the primary internalization of CV-B3, and DAF is a regulatory and attachment protein that acts as a co-receptor [27–32]. As shown in Fig. 2, all EC cell lines expressed moderate to high levels of DAF and CAR and are thus potential targets for CV-B3/2035A. Next, the susceptibility of the EC cell lines to lytic infection with CV-B3/2035A was evaluated in vitro. Cell monolayers were infected with CV-B3/2035A at different MOIs (10-fold series, from 10^− 3^ to 10) and incubated at 37 °C for 72 h. Microscopic examination revealed that infection by CV-B3/2035A at an MOI of 10 induced extensive lytic destruction of all three EC cell lines (Fig. 3a). Then, CCK8 assay was used to correlate CV-B3/2035A-mediated cytotoxicity with 10-fold viral input multiplicity (MOI) in infected EC cell monolayers. As shown in Fig. 3b, CV-B3/2035A induced > 50% cytotoxicity in HEC-1-B and Ishikawa cells at MOIs ≥10^− 3^, and in HEC-1-A cells at MOIs ≥10^− 2^, thus demonstrating potent oncolytic activity in all EC cell lines in vitro.

### Growth kinetics of CV-B3/2035A in human EC cells in vitro

Since replication of oncolytic virus in cancer cells typically results in a substantial amplification of the input viral load, which may facilitate oncolysis of cancer cells nearby or at distant sites of metastasis, the production of CV-B3/2035A progeny virus in the EC cell lines was further assessed by evaluating viral growth kinetics. EC cell monolayers were challenged with CV-B3/2035A at an MOI of 0.1, and viral replication was monitored for 48 h. As shown in Fig. 3c, similar viral growth kinetics were obtained in HEC-1-B and Ishikawa cells, with the exception of HEC-1-A cells, where viral growth kinetics was slower. All EC cell lines produced high virus yields (10^6^–10^8^ TCID50/well) at 48 h post-infection (hpi). Due to a slower growth kinetics, HEC-1-A cells had approximately 5-fold-less virus yields than HEC-1-B and Ishikawa cells. These data indicated that CV-B3/2035A initiated a robust productive infection in all EC cell lines in vitro.

### Oncolytic activity of CV-B3/2035A in human EC xenografts in nude mice in vivo

**Fig. 4:**
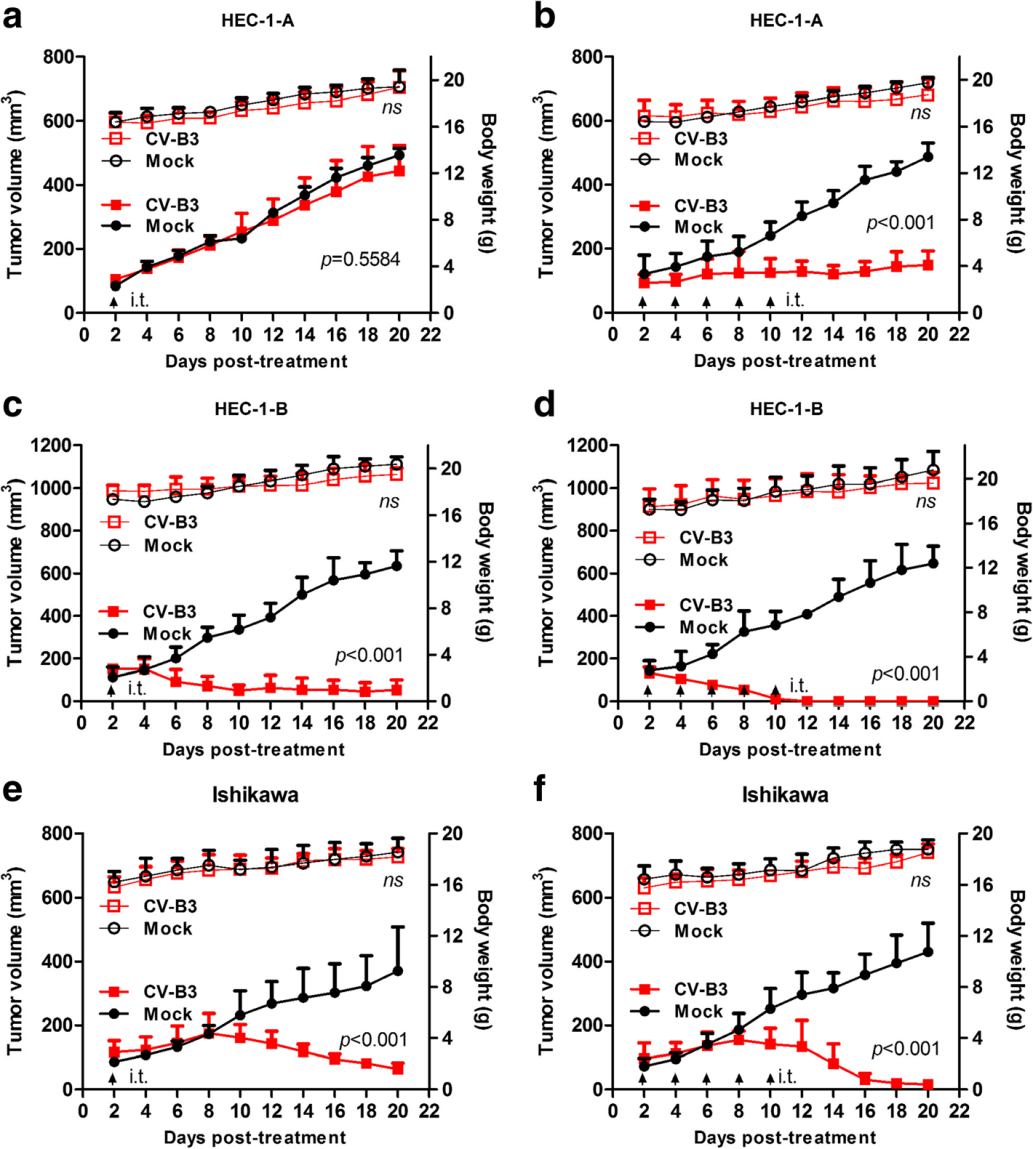
Direct oncolytic effect of intratumoral CV-B3/2035A administration on EC xenografts in vivo. Nude mice bearing HEC-1-A (**a, b**), HEC-1-B (**c, d**) and Ishikawa (**e, f**) xenografts were treated intratumorally (i.t.) with either a single or five consecutive doses of CV-B3/2035A (5 × 10^6^ TCID50 per dose) or PBS (Mock) (*n* = 5 per group). Tumor growth (solid symbols) and body weight (open symbols) of the tumor-bearing mice were monitored over a 20-day period. Statistical analysis was performed by two-way ANOVA. Data are shown in means ± SD. Black arrows indicate treatments; ns, not significant

To evaluate the in vivo oncolytic effect of CV-B3/2035A on human EC in vivo, nude mice models bearing EC subcutaneous xenografts were established. When tumor size reached ~ 0.5 cm in diameter, xenografts of three EC cell lines received either a single dose or five consecutive doses (5 × 10^6^ TCID50 per dose) of intratumoral CV-B3/2035A or PBS (*n* = 5 in each group), and tumor volumes were measured over a 20-day period. As shown in Fig. 4, intratumoral treatment with CV-B3/2035A resulted in dose-dependent inhibition of tumor growth, and the results were generally consistent with the in vitro data. A single dose of CV-B3/2035A administered into HEC-1-B and Ishikawa xenografts significantly suppressed their growth (Fig. 4c and e), whilst the same dose failed to inhibit the growth of HEC-1-A xenografts (Fig. 4a). On the other hand, five consecutive doses of CV-B3/2035A demonstrated potent anti-tumor effects on HEC-1-B and Ishikawa xenografts and caused complete tumor elimination in all CV-B3/2035A-treated mice (Fig. 4d and f); for HEC-1-A xenografts, the same doses resulted in significant tumor growth inhibition (Fig. 4b).

**Fig. 5:**
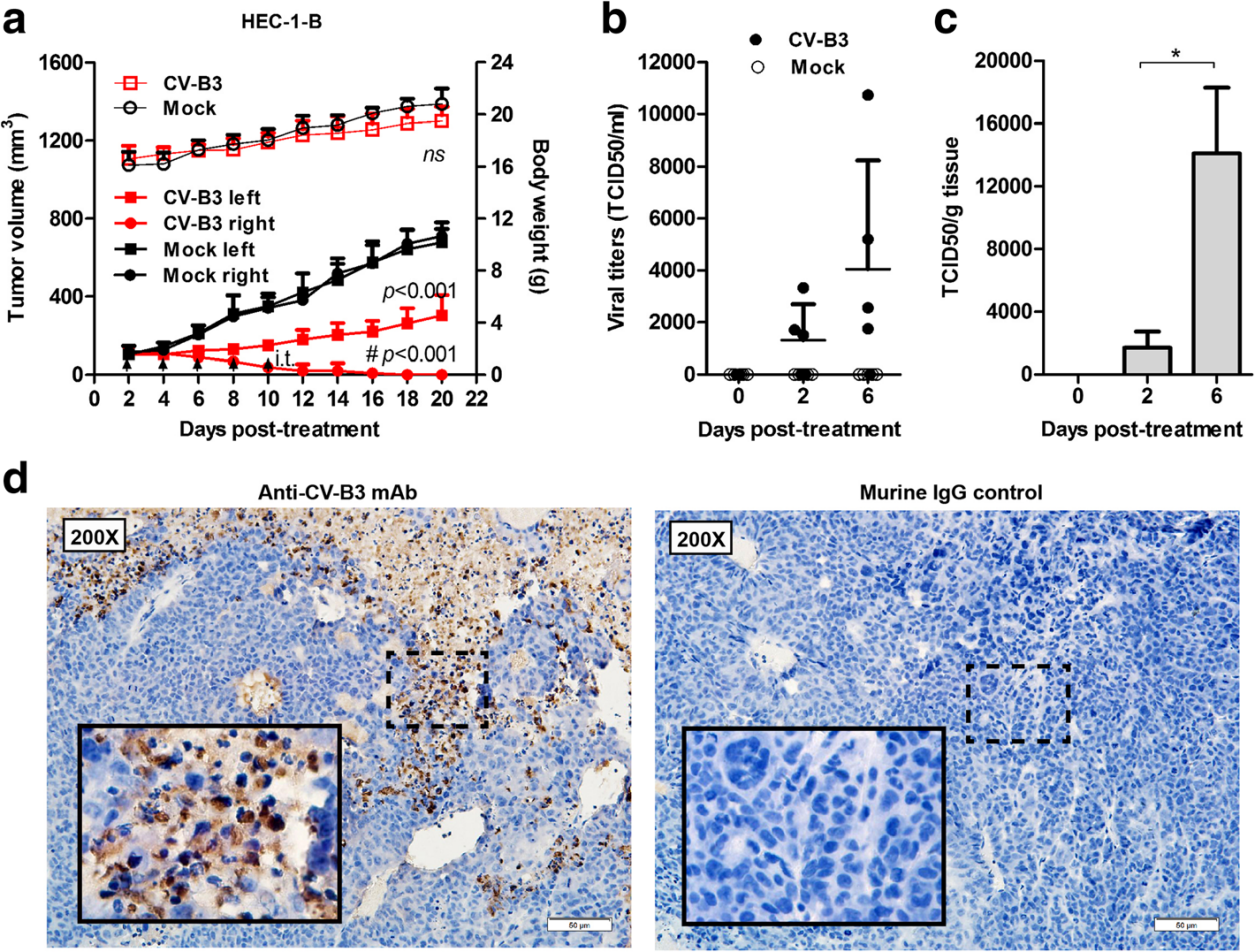
Indirect anti-tumor effect of intratumoral CV-B3/2035A administration on untreated contralateral EC xenografts in vivo*.* **a** Five consecutive doses of CV-B3/2035A (5 × 10^6^ TCID50 per dose) or PBS (Mock) were administered intratumorally (i.t.) into the right flank tumors in nude mice bearing bilateral HEC-1-B xenografts (*n* = 5 per group). Tumor growth (solid symbols) and body weight (open symbols) of the treated nude mice were monitored over a 20-day period. Statistical analysis was performed by two-way ANOVA. Data are shown in means ± SD. Each symbol represents the statistical significance of right (†) and left (#) lateral tumors between untreated and CV-B3/2035A-treated groups. Black arrows indicate treatments; ns, not significant. **b** Nude mice (*n* = 5 per group) were bled via the saphenous vein 1 h, 2 and 6 days post-treatment. Infectious CV-B3/2035A in the serum was determined by TCID50 assay on RD cells. The viremia levels (TCID50/ml) are shown in means ± SD. **c** Untreated contralateral HEC-1-B xenografts (*n* = 3) were collected from nude mice 1 h, 2 and 6 days post-treatment. Tumor samples were cut into pieces and homogenized with a Dounce tissue grinder (Sigma-Aldrich, USA) in ice-cold MEM, followed by a freeze-thaw protocol to release virus from cells. Clarified (centrifugation) supernatants were used to perform TCID50 assay on RD cells. Virus titers (TCID50/g tissue) are shown in means ± SD. *p < 0.05. **d** Tissue sections of untreated contralateral HEC-1-B xenografts from nude mice 20 days post-treatment were immunostained using antibodies specific for CV-B3 or murine IgG control. The inset boxes represent higher magnification images of the areas outlined by the dotted lines in the two panels

Next, a nude mouse model bearing bilateral subcutaneous HEC-1-B xenografts was used to investigate possible systemic oncolytic effects of CV-B3/2035A against EC. As shown in Fig. 5a, five consecutive administrations (5 × 10^6^ TCID50 per dose) of CV-B3/2035A into the right flank tumors significantly suppressed their growth until total tumor elimination; meanwhile, the distant untreated xenografts in the left flank showed significant tumor volume reduction when compared with the mock group (*n* = 5 in each group). The levels of circulating infectious virus in the bloodstream were determined by TCID50 assay on RD cells at 1 h after the first treatment (day 2) and at 2 (day 4) and 6 days (day 8) post-treatment, and the results suggested that viremia occurred in nude mice treated with intratumoral administration of CV-B3/2035A (Fig. 5b). Additionally, virus became detectable in the contralateral tumors at 2 days post-treatment, and the titers significantly increased at 6 days post-treatment (Fig. 5c), which may reflect viral accumulation and replication. Moreover, on day 20, abundant CV-B3 antigens were identified in areas distributed throughout the untreated contralateral tumors by IHC analysis (Fig. 5d). These CV-B3-antigen-positive areas also showed obvious tissue damage, while structures of the surrounding uninfected tissues remained intact. These data indicated that CV-B3/2035A exerted an indirect anti-tumor effect on contralateral tumors, probably via viremia.

**Fig. 6:**
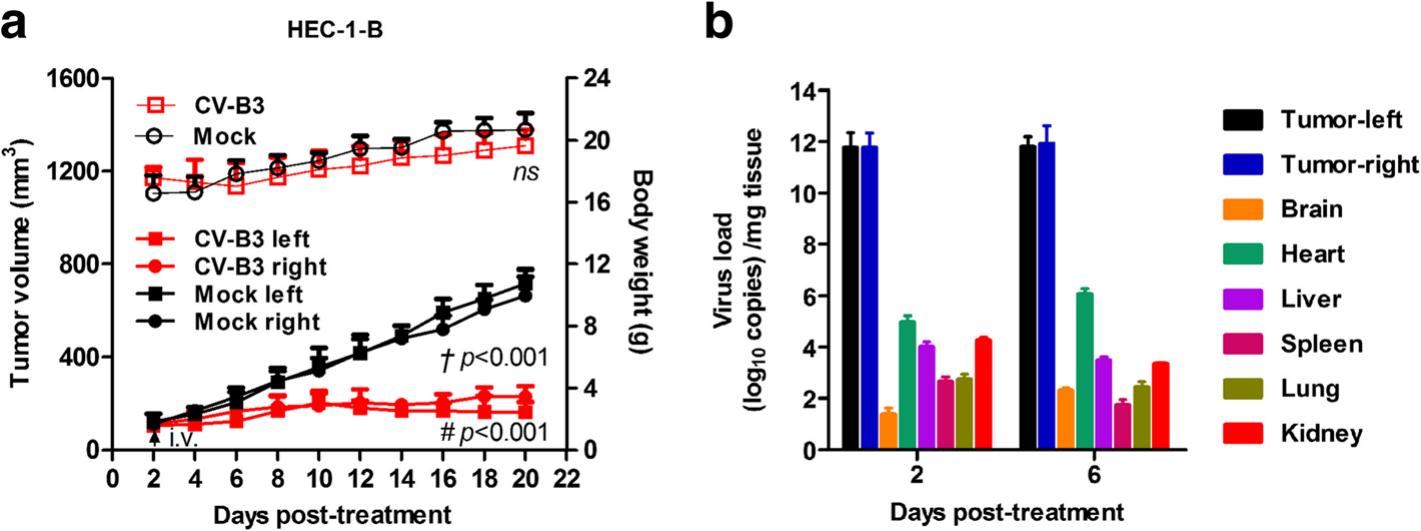
Oncolytic effect of intravenous CV-B3/2035A administration on EC xenografts in vivo. **a** A single dose (1 × 10^6^ TCID50) of CV-B3/2035A or PBS (Mock) was administered intravenously (i.v.) into the nude mice bearing bilateral HEC-1-B xenografts (*n* = 5 per group). Tumor growth (solid symbols) and body weight (open symbols) of nude mice were monitored over a 20-day period. Statistical analysis was performed by two-way ANOVA. Data are shown in means ± SD. The symbols, † and #, represent the statistical significance of right and left lateral tumors between untreated and CV-B3/2035A-treated groups, respectively. Black arrows indicate treatments; ns, not significant. **b** Biodistribution of intravenously delivered CV-B3/2035A in tumor-bearing nude mice was analyzed at 2 and 6 days post-treatment by qRT-PCR. Data are shown in means ± SD (*n* = 3 per group)

To further determine the systemic anti-tumor activity, in vivo selectivity and potential toxicity of CV-B3/2035A, nude mice with bilateral HEC-1-B xenografts were treated with a single intravenous dose of 1 × 10^6^ TCID50 of CV-B3/2035A. As shown in Fig. 6a, a single intravenous CV-B3/2035A injection directly caused significant and similar inhibition of tumor growth in both flanks of nude mice when compared with the mock group (*n* = 5 in each group). Biodistribution of viral RNA was analyzed at 2 (day 4) and 6 days (day 8) post-treatment by qRT-PCR, and the results showed that CV-B3/2035A was much more enriched in HEC-1-B tumor xenografts than in any other tissue (Fig. 6b; *n* = 3). On day 20, histological analysis of vital tissues of CV-B3/2035A-treated and untreated mice, including brain, heart, liver, spleen, lung and kidney, were performed by H&E staining, and no obvious pathological changes were observed at this time point (Additional file 1: Figure S1). Additionally, there was no nude mice died and no significant difference in mean body weight between control and CV-B3/2035A-treated groups during the course of the in vivo study (Figs. 4, 5a and 6a).

### Oncolytic activity of CV-B3/2035A in patient-derived EC samples ex vivo

**Fig. 7:**
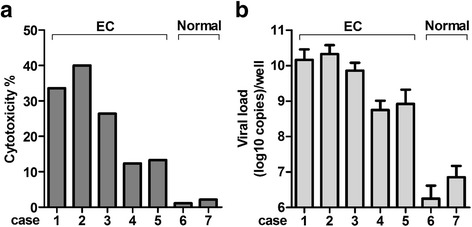
Oncolytic effect of CV-B3/2035A on ex vivo patient-derived endometrioid adenocarcinoma samples. Five endometrioid adenocarcinoma samples (EC; Case 1–5) and two normal endometrium samples (Case 6–7) were divided into ∼1-mm^3^ pieces and treated with CV-B3/2035A (1 × 10^6^ TCID50) or left untreated (*n* = 4 per group). After 72 h, **a** tissue viability was assessed using a modified HDRA method and **b** replication of CV-B3/2035A in both tumor and normal tissue samples was evaluated by qRT-PCR

To estimate the possible clinical efficacy of CV-B3/2035A in the treatment of human EC, biopsied or surgical specimens of primary human EC from 5 patients (Case 1–5 in Fig. 7; sample information shown in Table 2) and 2 normal human endometrium samples (Case 6 and 7 in Fig. 7) were collected for testing CV-B3/2035A–mediated oncolysis. Tumor or normal tissues slices (~ 1 mm^3^) were either infected with 1 × 10^6^ TCID50 per well of CV-B3/2035A or left untreated (*n* = 4 in each group), and viability of the tissue slices was assayed 72 h later. As shown in Fig. 7a, CV-B3/2035A-treatment caused a 10–40% loss in viability of the patient-derived EC tissues but caused little damage to normal endometria tissues when compared with the untreated groups. Subsequently, replication of CV-B3/2035A in both tumor and normal tissue samples was evaluated by qRT-PCR. The results showed that CV-B3/2035A produced much higher levels of viral RNA (Fig. 7b) in the patient-derived EC tissues than in normal endometria tissues (*n* = 4 in each group). These data could further support the potential therapeutic use of CV-B3/2035A against EC.

**Table 2:** Patient-derived endometrial cancer samples used in this study

Patient No.	Age	Disease	Tumor stage
1	45	Endometrioid adenocarcinoma	Stage IA, grade 2
2	59		Stage II, grade 2
3	42		Stage IIIA, grade 1
4	55		Stage IIIC, grade 1
5	54		Stage IIIC, grade 1

## Discussion

Malignancies resistant to current treatments require novel therapeutic approaches. A promising option is oncolytic virotherapy with picornaviruses, which have shown encouraging results in recent clinical trials, including coxsackievirus A21 (CV-A21; CAVATAK), poliovirus (PVS-RIPO) and Seneca Valley virus (NTX-010) (reviewed in [33]). Recently, CV-B3 (prototype Nancy strain) has been found to have natural oncolytic activity against a variety of human tumor cell types, especially in NSCLC cell lines [21]. These observations indicated that CV-B3 strains may have broad-spectrum antitumor effects and thus warrant further investigation.

EC is one of the most common gynecological malignancies and its incidence is rising among pre- and postmenopausal women globally. Although the outcome of standard treatment of early-stage EC by surgery is generally favorable, available treatment options for advanced, recurrent and metastatic ECs remain very limited [3]. Oncolytic virotherapy as an emerging immunotherapeutic modality for cancer therapy may improve outcomes in EC, but there have been only few reports about oncolytic viruses for EC treatment in recent years [34, 35]. In this context, the aim of the present study was to investigate the potential of a CV-B3 strain 2035A as a novel viral therapeutic agent against EC.

CV-B3/2035A was isolated in China and possessed oncolytic ability in a wide range of human tumor cells (Additional file 1: Table S2). In vitro assays demonstrated that all examined EC cell lines expressed CV-B3 receptors (DAF and CAR) (Fig. 2) and were markedly susceptible to CV-B3/2035A-induced cytotoxicity (Fig. 3). Previous studies have shown that CAR and DAF are expressed on EC cells of different histological types, grades and stages [32, 33]. CAR expression was reported to be increased with increasing grade of tumor in EC patients [32], while DAF expression is upregulated mainly on well-differentiated early-stage EC cells [33]. These findings suggest that EC may be a good target for CV-B3/2035A.

EC relapses both locally and at distant sites. Therefore, in nude mice models bearing subcutaneous EC xenografts, the in vivo therapeutic efficacy for both intratumoral and systemic administrations of CV-B3/2035A was evaluated. Firstly, the results demonstrated that the ability of CV-B3/2035A to inhibit tumor growth varied in different EC cell lines (Fig. 4). The growth inhibition by CV-B3/2035A on HEC-1-B and Ishikawa xenografts was marked and sustained until day 42 (the actual time of study termination; data not shown). In contrast, HEC-1-A xenografts were much less sensitive to growth inhibition by CV-B3/2035A, which may be explained by differences in the level of surface CAR expression (Fig. 2) or by other factors that help to define the viral tropism. Additionally, viremia was observed following intratumoral administration of CV-B3/2035A into the EC xenografts, which was probably the cause of viral accumulation and replication in the distant untreated tumors (Fig. 5b). Furthermore, IHC analysis demonstrated that CV-B3/2035A spread throughout the contralateral tumors by disrupting the tumor architecture, which could lead to their growth inhibition (Fig. 5c). As expected, a single intravenous injection of CV-B3/2035A that mimicked the viremia exerted significant therapeutic effects on the EC xenografts, thus supporting its systemic use (Fig. 6a). Besides the EC xenograft/nude mice model, CV-B3/2035A also exhibited therapeutic efficacy in patient-derived EC samples ex vivo (Fig. 7). Altogether, these data indicated that CV-B3/2035A could be effective for both local and systemic treatment of EC.

A preliminary toxicity assessment was conducted in nude mice bearing EC xenografts following a single intravenous injection of CV-B3/2035A. Neither death nor significant body weight loss was observed in the tumor-bearing mice throughout the study. Biodistribution analysis showed that CV-B3/2035A had high tumor selectivity in the EC xenograft/nude mice model (Fig. 6b). Except for the EC xenografts, viral RNA was mainly detected in the heart of nude mice. However, no cardiac pathology was found in any examined mouse (*n* = 5) by histological analysis on day 20 (Additional file 1: Figure S1). To date, a number of CV-B3 strains, including CV-B3/Nancy, CV-B3/MKP, CV-B3/20 and CV-B3/28, are known to induce viral myocarditis in nude mice [36–38]. On the other hand, some CV-B3 strains, including CV-B3/0, CV-B3/GA and CV-B3/CO, are non-cardiovirulent [38, 39]. These data indicated that CV-B3/2035A, isolated from a case of mild HFMD, may be a benign non-cardiovirulent strain; nonetheless, further studies are warranted to test this hypothesis.

Even if CV-B3/2035A does not cause systemic disease in mice, its direct use may still cause serious diseases, such as myocarditis, aseptic meningitis and encephalitis, in humans. Therefore, there is a need for genetic modification of CV-B3/2035A to reduce its cytotoxicity to normal tissues while retaining oncolytic properties. Recently, microRNAs (miRNA) have been exploited to regulate tropisms of replication-competent oncolytic viruses by insertion of miRNA target (miRT) sequences into viral genomes (reviewed in [40]). Such miRNA-based genetic modulation has proven an effective method to attenuate the cytotoxic effect of CV-B3 without affecting its antitumor activity in NSCLC cells and thus could overcome a major hurdle to clinical development of this virotherapeutics candidate [41, 42]. Therefore, by combining with miRNA regulation, CV-B3/2035A could be further improved in safety. Furthermore, since CV-B3 has been extensively studied as a recombinant viral vector [43–45], CV-B3/2035A could be further improved in oncolytic potency by inserting anti-tumor genes into the viral genome. In addition, future research may be needed to explore the synergies between virotherapy with CV-B3/2035A and small molecular anticancer compounds or immune checkpoint inhibitors in EC treatment.

## Conclusions

In summary, CV-B3 2035A strain exhibited effective oncolytic activities in human EC cell lines both in vitro and in vivo as well as in patient-derived EC samples ex vivo. Future studies will be necessary to determine the mechanisms underlying the oncolytic effects of CV-B3/2035A in EC cells and to improve the safety and potency of CV-B3/2035A as a potential virotherapy agent for EC treatment.

## Additional file

Additional file 1: **Table S1.** Primer and probe sequences used for qRT-PCR. **Table S2.** In vitro oncolytic activity of CV-B3/2035A in a panel of human tumor cell lines. **Figure S1.** Representative H&E-stained histologic images of vital tissues (including brain, heart, liver, spleen, lung and kidney) of CV-B3/2035A-treated and untreated mice (CTL) euthanized on day 20. (PDF 313 kb)

## Abbreviations

CAR: Coxsackievirus and adenovirus receptor
CV-B3: Coxsackievirus B3
DAF: Decay accelerating factor
EC: Endometrial cancer
HDRA: Histoculture drug response assay
HFMD: Hand, foot and mouse disease
NSCLC: Non–small cell lung cancer

## References

[1] Siegel RL, Miller KD, Jemal A (2017). Cancer statistics, 2017. CA Cancer J Clin.

[2] Bokhman JV (1983). Two pathogenetic types of endometrial carcinoma. Gynecol Oncol.

[3] Morice P, Leary A, Creutzberg C, Abu-Rustum N, Darai E (2016). Endometrial cancer. Lancet.

[4] McAlpine JN, Temkin SM, Mackay HJ (2016). Endometrial cancer: not your grandmother's cancer. Cancer.

[5] Lajer H, Elnegaard S, Christensen RD, Ortoft G, Schledermann DE, Mogensen O (2012). Survival after stage IA endometrial cancer; can follow-up be altered? A prospective nationwide Danish survey. Acta Obstet Gynecol Scand.

[6] Janda M, Gebski V, Brand A, Hogg R, Jobling TW, Land R, Manolitsas T, McCartney A, Nascimento M, Neesham D (2010). Quality of life after total laparoscopic hysterectomy versus total abdominal hysterectomy for stage I endometrial cancer (LACE): a randomised trial. Lancet Oncol.

[7] Kilgore LC, Partridge EE, Alvarez RD, Austin JM, Shingleton HM, Noojin F, Conner W (1995). Adenocarcinoma of the endometrium - survival comparisons of patients with and without pelvic node sampling. Gynecol Oncol.

[8] Kitchener H, Swart AM, Qian Q, Amos C, Parmar MK, Group As (2009). Efficacy of systematic pelvic lymphadenectomy in endometrial cancer (MRC ASTEC trial): a randomised study. Lancet.

[9] Kendrick JE, Huh WK (2007). Treatment considerations in advanced endometrial cancer. Curr Oncol Rep.

[10] Homesley HD, Filiaci V, Gibbons SK, Long HJ, Cella D, Spirtos NM, Morris RT, DeGeest K, Lee R, Montag A (2009). A randomized phase III trial in advanced endometrial carcinoma of surgery and volume directed radiation followed by cisplatin and doxorubicin with or without paclitaxel: a gynecologic oncology group study. Gynecol Oncol.

[11] Randall ME, Filiaci VL, Muss H, Spirtos NM, Mannel RS, Fowler J, Thigpen JT, Benda JA (2006). Gynecologic Oncology Group S: randomized phase III trial of whole-abdominal irradiation versus doxorubicin and cisplatin chemotherapy in advanced endometrial carcinoma: a Gynecologic Oncology Group Study. J Clin Oncol.

[12] Kokka F, Brockbank E, Oram D, Gallagher C, Bryant A. Hormonal therapy in advanced or recurrent endometrial cancer. Cochrane Database Syst Rev. 2010:CD007926. 10.1002/14651858.CD007926.pub2 PMC4164823 21154390

[13] Gadducci A, Cosio S, Genazzani AR (2006). Old and new perspectives in the pharmacological treatment of advanced or recurrent endometrial cancer: hormonal therapy, chemotherapy and molecularly targeted therapies. Crit Rev Oncol Hematol.

[14] Maggi R, Lissoni A, Spina F, Melpignano M, Zola P, Favalli G, Colombo A, Fossati R (2006). Adjuvant chemotherapy vs radiotherapy in high-risk endometrial carcinoma: results of a randomised trial. Br J Cancer.

[15] Garcia AA, Blessing JA, Nolte S, Mannel RS, Gynecologic Oncology G (2008). A phase II evaluation of weekly docetaxel in the treatment of recurrent or persistent endometrial carcinoma: a study by the gynecologic oncology group. Gynecol Oncol.

[16] Muggia FM, Blessing JA, Sorosky J, Reid GC (2002). Phase II trial of the pegylated liposomal doxorubicin in previously treated metastatic endometrial cancer: a gynecologic oncology group study. J Clin Oncol.

[17] Keller BA, Bell JC (2016). Oncolytic viruses-immunotherapeutics on the rise. J Mol Med (Berl).

[18] Kaufman HL, Kohlhapp FJ, Zloza A (2015). Oncolytic viruses: a new class of immunotherapy drugs. Nat Rev Drug Discov.

[19] Liu BL, Robinson M, Han ZQ, Branston RH, English C, Reay P, McGrath Y, Thomas SK, Thornton M, Bullock P (2003). ICP34.5 deleted herpes simplex virus with enhanced oncolytic, immune stimulating, and anti-tumour properties. Gene Ther.

[20] Kohlhapp FJ, Kaufman HL (2016). Molecular pathways: mechanism of action for Talimogene Laherparepvec, a new oncolytic virus immunotherapy. Clin Cancer Res.

[21] Miyamoto S, Inoue H, Nakamura T, Yamada M, Sakamoto C, Urata Y, Okazaki T, Marumoto T, Takahashi A, Takayama K (2012). Coxsackievirus B3 is an oncolytic virus with immunostimulatory properties that is active against lung adenocarcinoma. Cancer Res.

[22] Reed LJ, Muench H (1938). A simple method of estimating fifty per cent endpoints. Am J Hyg.

[23] Wang W, Wang X, Yang L, Fu W, Pan D, Liu J, Ye J, Zhao Q, Zhu H, Cheng T, Xia N (2016). Modulation of host CD59 expression by varicella-zoster virus in human xenografts in vivo. Virology.

[24] Lee SY, Jeon DG, Cho WH, Song WS, Kim MB, Park JH (2006). Preliminary study of chemosenstivity tests in osteosarcoma using a histoculture drug response assay. Anticancer Res.

[25] Kong X, Xu X, Yan Y, Guo F, Li J, Hu Y, Zhou H, Xun Q (2014). Estrogen regulates the tumour suppressor MiRNA-30c and its target gene, MTA-1, in endometrial cancer. PLoS One.

[26] Haughian JM, Bradford AP (2009). Protein kinase C alpha (PKCalpha) regulates growth and invasion of endometrial cancer cells. J Cell Physiol.

[27] Carson SD (2001). Receptor for the group B coxsackieviruses and adenoviruses: CAR. Rev Med Virol.

[28] Carson SD, Chapman NM, Hafenstein S, Tracy S (2011). Variations of coxsackievirus B3 capsid primary structure, ligands, and stability are selected for in a coxsackievirus and adenovirus receptor-limited environment. J Virol.

[29] Pan J, Zhang L, Organtini LJ, Hafenstein S, Bergelson JM (2015). Specificity of coxsackievirus B3 interaction with human, but not murine, decay-accelerating factor: replacement of a single residue within short consensus repeat 2 prevents virus attachment. J Virol.

[30] Hafenstein S, Bowman VD, Chipman PR, Bator Kelly CM, Lin F, Medof ME, Rossmann MG (2007). Interaction of decay-accelerating factor with coxsackievirus B3. J Virol.

[31] Tomko RP, Xu R, Philipson L (1997). HCAR and MCAR: the human and mouse cellular receptors for subgroup C adenoviruses and group B coxsackieviruses. Proc Natl Acad Sci U S A.

[32] Shafren DR, Williams DT, Barry RD (1997). A decay-accelerating factor-binding strain of coxsackievirus B3 requires the coxsackievirus-adenovirus receptor protein to mediate lytic infection of rhabdomyosarcoma cells. J Virol.

[33] Masemann D, Boergeling Y, Ludwig S (2017). Employing RNA viruses to fight cancer: novel insights into oncolytic virotherapy. Biol Chem.

[34] Liu YP, Wang J, Avanzato VA, Bakkum-Gamez JN, Russell SJ, Bell JC, Peng KW (2014). Oncolytic vaccinia virotherapy for endometrial cancer. Gynecol Oncol.

[35] Liu YP, Steele MB, Suksanpaisan L, Federspiel MJ, Russell SJ, Peng KW, Bakkum-Gamez JN (2014). Oncolytic measles and vesicular stomatitis virotherapy for endometrial cancer. Gynecol Oncol.

[36] Lee C, Maull E, Chapman N, Tracy S, Wood J, Gauntt C (1997). Generation of an infectious cDNA of a highly cardiovirulent coxsackievirus B3(CVB3m) and comparison to other infectious CVB3 cDNAs. Virus Res.

[37] Liu B, Li Z, Xiang F, Li F, Zheng Y, Wang G (2014). The whole genome sequence of coxsackievirus B3 MKP strain leading to myocarditis and its molecular phylogenetic analysis. Virol J.

[38] Tracy S, Drescher KM, Chapman NM, Kim KS, Carson SD, Pirruccello S, Lane PH, Romero JR, Leser JS (2002). Toward testing the hypothesis that group B coxsackieviruses (CVB) trigger insulin-dependent diabetes: inoculating nonobese diabetic mice with CVB markedly lowers diabetes incidence. J Virol.

[39] Dunn JJ, Chapman NM, Tracy S, Romero JR (2000). Genomic determinants of cardiovirulence in coxsackievirus B3 clinical isolates: localization to the 5′ nontranslated region. J Virol.

[40] Ruiz AJ, Russell SJ (2015). MicroRNAs and oncolytic viruses. Curr Opin Virol.

[41] Miyamoto S, Inoue H, Sagara M, Wang BB, Takayama K, Shimizu H, Nakanishi Y, Tani K (2013). Dual microRNA-regulated oncolytic Coxsackievirus B3 infection displays antitumor activity with attenuated pathogenicity in mice. Mol Ther.

[42] Miyamoto S, Sagara M, Kohara H, Tani K (2017). Oncolytic coxsackievirus therapy as an immunostimulator. Rinsho Ketsueki.

[43] Chapman NM, Kim KS, Tracy S, Jackson J, Hofling K, Leser JS, Malone J, Kolbeck P (2000). Coxsackievirus expression of the murine secretory protein interleukin-4 induces increased synthesis of immunoglobulin G1 in mice. J Virol.

[44] Lim BK, Shin JO, Lee SC, Kim DK, Choi DJ, Choe SC, Knowlton KU, Jeon ES (2005). Long-term cardiac gene expression using a coxsackieviral vector. J Mol Cell Cardiol.

[45] Hofling K, Tracy S, Chapman N, Kim KS, Smith Leser J (2000). Expression of an antigenic adenovirus epitope in a group B coxsackievirus. J Virol.

