# Supplementary material for: Oncolytic activity of a coxsackievirus B3 strain in human endometrial cancer cell lines
Source: Virol J. 2018 Apr 10;15:65. doi: 10.1186/s12985-018-0975-x (PMC5891967; doi:10.1186/s12985-018-0975-x)
Supplement: Supplementary file 1 — Table S1. Primer and probe sequences used for qRT-PCR. Table S2. In vitro oncolytic activity of CV-B3/2035A in a panel of human tumor cell lines. Figure S1. Representative H&E-stained histologic images of vital tissues (including brain, heart, liver, spleen, lung and kidney) of CV-B3/2035A-treated and untreated mice (CTL) euthanized on day 20. (PDF 313 kb) [file 12985_2018_975_MOESM1_ESM.pdf]

Table S1. Primer and probe sequences used for qRT-PCR

Primer or probe	Sequence (5'→3')	Nucleotide Position (range)
Forward primer	TCCTCCGGCCCCTGA	447-461
Reverse primer	AATTGTCACCATAAGCAGCCA	602-582
Probe	FAM-CGGAACCGACTACTTTGGGTGTCCGT-BHQ1	536-561

Table S2. *In vitro* oncolytic activity of CV-B3/2035A in a panel of human tumor cell lines. Various human tumor cell lines and normal MRC-5 and HFF-1 cells were infected with CV-B3/2035A at MOIs of 10, 1, 0.1 and 0.01. At 72 hours post-infection, cell viability was assessed by CCK8 assay. "++", "+" and "-" indicate greater than 50% cytotoxicity, less than 50% cytotoxicity and no cytotoxicity, respectively.

MOI / CELL LINE	10	1	0.1	0.01
**RD**	++	++	++	+
**SW1116**	++	++	+	-
**SW480**	++	++	++	++
**HT29**	++	++	++	++
**AGS**	++	++	++	+
**SGC7901**	++	++	++	+
**BGC823**	++	++	++	++
**NCI-N87**	++	++	++	-
**TE-1**	++	+	-	-
**DMS114**	++	++	++	++
**SPC-A-1**	++	++	++	++
**NCI-H1975**	++	++	-	-
**NCI-H1299**	++	++	++	+
**A549**	++	++	+	+
**NCI-H661**	++	++	++	++
**EBC-1**	++	++	++	+
**NCI-H1703**	++	++	+	+
**C3A**	++	++	++	++
**HEPG2**	++	++	++	+
**SMMC7721**	++	++	++	++
**BEL7404**	++	++	++	++
**BEL7402**	++	++	++	++
**HUH7**	++	++	++	++
**PLC/PRF/5**	++	++	++	++
**SKOV3**	++	++	+	-
**CAOV3**	++	++	+	-

**HEC-1-A**	++	++	+	-
**HEC-1-B**	++	++	++	++
**ISHIKAWA**	++	++	++	++
**HELA**	++	++	++	++
**CASKI**	++	++	++	++
**C-33A**	++	++	++	++
**SK-MEL-1**	++	++	+	+
**MEWO**	++	-	-	-
**BCAP37**	++	++	++	++
**BT-474**	++	++	++	++
**MDA-MB-231**	++	+	+	-
**A498**	++	++	++	+
**786-O**	+	+	-	-
**CAPAN-2**	++	-	-	-
**HPAF-2**	++	++	+	+
**U2OS**	++	++	++	-
**DU145**	++	++	++	+
**LNCAP**	++	++	++	+
**SH-SY5Y**	++	++	++	++
**CNE**	++	++	+	-
**RPMI 2650**	++	++	+	+
**HEP-2**	++	++	++	++
**TT**	++	++	++	-
**J82**	++	+	-	-
**5637**	++	++	+	-
**DAUDI**	++	++	+	-
**RAJI**	++	-	-	-
**MRC-5**	-	-	-	-
**HFF-1**	-	-	-	-

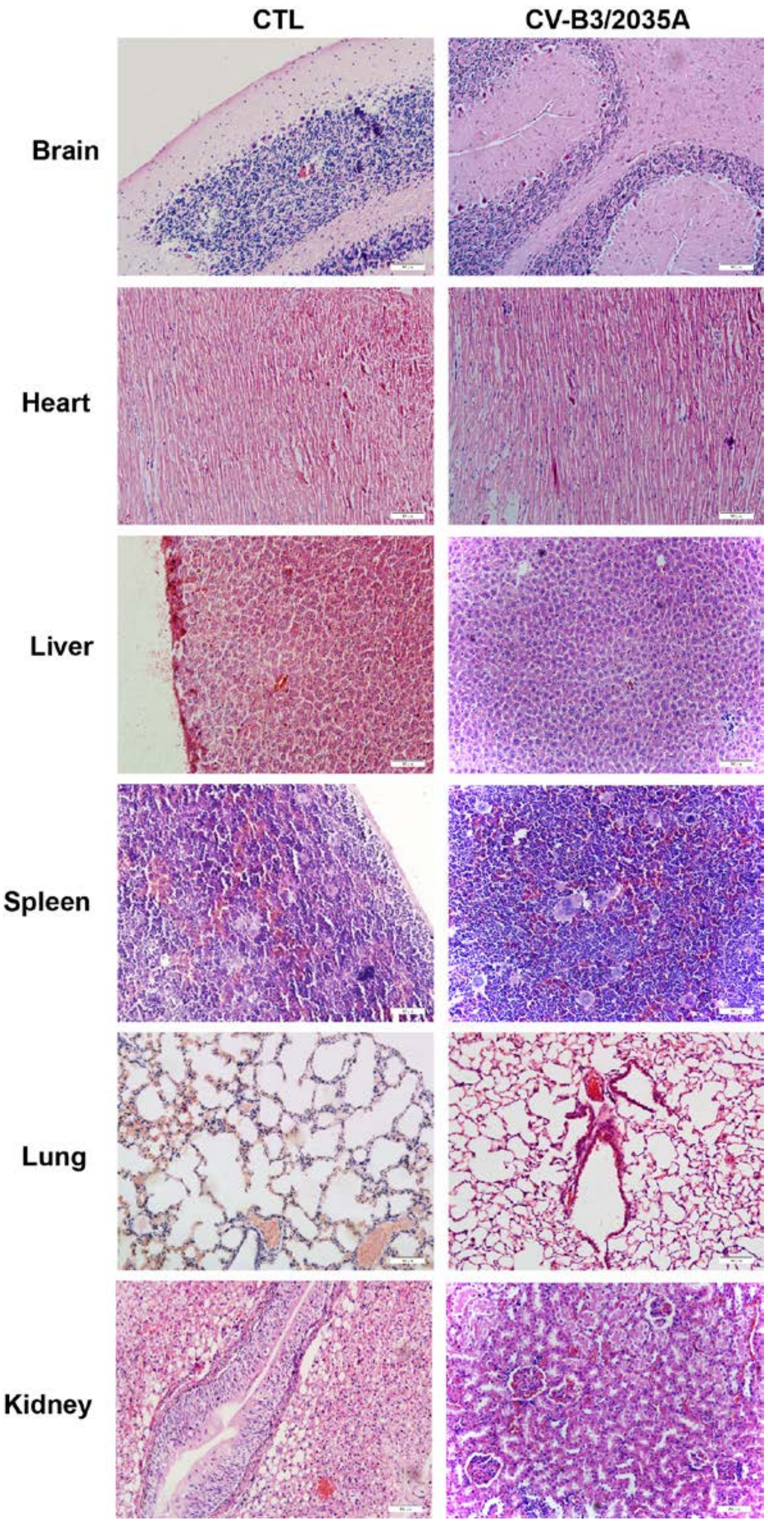

Figure S1. Representative hematoxylin and eosin (H&E)-stained histologic images of vital tissues (including brain, heart, liver, spleen, lung and kidney) of CV-B3/2035A-treated and untreated mice (CTL) euthanized on day 20. No obvious pathological changes were observed.
